# Twig-Like Middle Cerebral Artery Anomaly Mimicking Vaso-Occlusion Disease: A Rare Neuroimaging Finding

**DOI:** 10.7759/cureus.86883

**Published:** 2025-06-27

**Authors:** Andres Felipe Rios Victoria, Eduardo Trejo Olguin, Ana Karen Luna Marroquin, Alan Giresse Lozano Alanis, Arturo Maximiliano Rodriguez Saldivar, Mariana Mercado Flores, Jesus Alberto Morales Gomez, Mario Alberto Campos Coy

**Affiliations:** 1 Neuroradiology, Hospital Universitario Dr. Jose Eleuterio Gonzalez, Monterrey, MEX; 2 Neurosurgery, Hospital Universitario Dr. Jose Eleuterio Gonzalez, Monterrey, MEX; 3 Head and Neck Radiology, Hospital Universitario Dr. Jose Eleuterio Gonzalez, Monterrey, MEX

**Keywords:** angiography, congenital anomaly, cta, rete middle cerebral artery, twig-like mca

## Abstract

Rete MCA, or the twig-like middle cerebral artery (tMCA), is a rare congenital vascular malformation that can mimic vaso-occlusive processes in advanced imaging. We present the case of a 52-year-old man who developed weakness in his left arm and neck pain due to a wound after being beaten by individuals. Non-contrast computed tomography (CT) was used to assess the cervical and craniofacial injuries. CT angiography showed a plexiform vascular network supplying the area of the M1 segment of the right MCA, with no evidence of remote collateral circulation or cerebral infarction. Our case was further delineated using digital subtraction angiography, and it revealed a non-progressive twig-like lesion on the right MCA. The patient was treated conservatively. This case points to the potential for a twig-like MCA to mimic vascular emergencies and re-emphasizes the importance of recognizing a tMCA as an innocuous vascular variant that can mimic pathological processes, which may change the interpretation of any future advanced imaging and treatment options.

## Introduction

The middle cerebral artery (MCA) is a direct branch of the internal carotid artery. It is the largest and most complex of the cerebral arteries and also serves as a collateral for the anterior cerebral artery (ACA) [1]. The MCA begins to form around 30 days into embryonic development and is responsible for providing blood supply to most of the cerebral neocortex. If the development of the MCA is disrupted due to unknown mechanisms or external factors, it may lead to fusion issues in the main trunk of the artery [2].

So far, variations of the MCA have been reported in 0.17%-4% of cases in angiographic and postmortem studies [3]. tMCA, also referred to as twig-like, aplastic, or unfused, is a rare cerebrovascular malformation with an incidence of less than 1.17% [4,5]. This malformation may be linked to neurological symptoms, asymptomatic patients, or may be discovered incidentally during radiological evaluations, often accompanied by hemorrhage and aneurysms due to the presence of fragile, plexiform vessels with immature arterial walls [6,7]. Currently, cerebral angiography is regarded as the gold standard for diagnosing such conditions. It is crucial to recognize the twig-like characteristics of the MCA to prevent misdiagnosis and unnecessary revascularization procedures.

This case involves a 52-year-old male patient who experienced head and neck trauma. Upon examination using computed tomography (CT), he was diagnosed with tMCA.

## Case presentation

**Figure 1:**
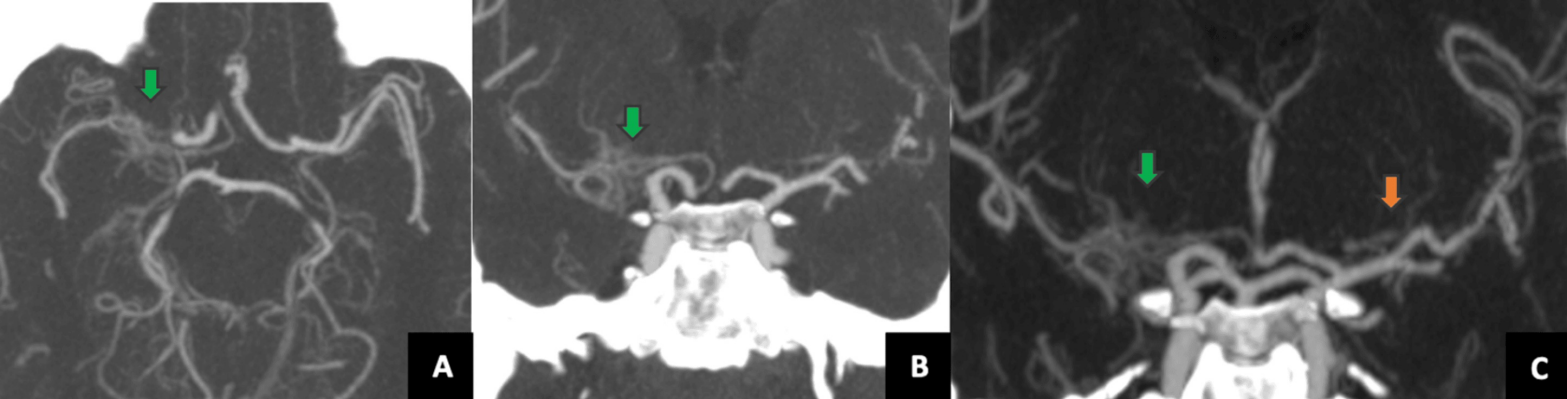
CT angiography in axial (A) and coronal planes (B and C) and maximum intensity projection (MIP) The CT angiography revealed an abnormal plexiform network (A and B) in the right M1 segment (green arrow) and early division of the left middle cerebral artery with trifurcation (C, orange arrow).

A 52-year-old man, previously asymptomatic, arrived at the emergency department with a wound on the left side neck and left arm pain after being beaten and injured by individuals. He was neurologically stable, with a National Institutes of Health Stroke Scale (NIHSS) score of 0. Upon his arrival, initial brain CT showed no infarct or hemorrhage (Figure 1, Panels A and B). CT angiography of the head and neck revealed an abnormal plexiform network in the right M1 segment and early division of the left MCA with trifurcation of the right MCA as an anatomic variant (Figure 1, Panel C).

**Figure 2:**
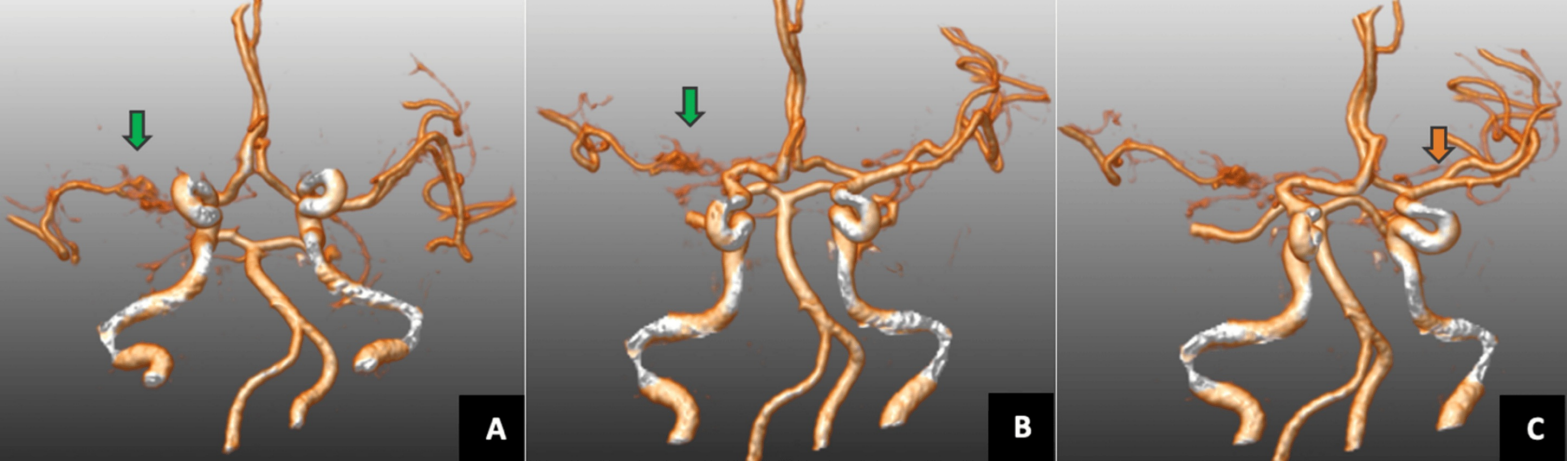
CT brain 3D reconstruction It showed an abnormal plexiform network in the right M1 segment (A and B, green arrow) and early division of the left middle cerebral artery with trifurcation (C, orange arrow).

CT brain 3D reconstruction showed an abnormal plexiform network in the right M1 segment (Figure 2, Panels A and B) and early division of the left MCA with trifurcation (Figure 2, Panel C).

**Figure 3:**
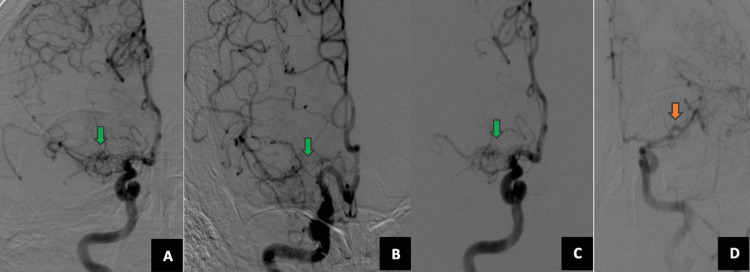
Digital subtraction angiography (DSA) DSA confirmed a twig-like anomaly on the right side without evidence of collateral circulation (A-C) and early division on the left middle cerebral artery (D).

The patient was treated conservatively for his neck wounds. Digital subtraction angiography was conducted to rule out hidden hemorrhages, vaso-occlusion, or complications of the malformation. A twig-like anomaly was confirmed on the right side without evidence of collateral circulation (Figure 3, Panels A-C) and early division on the left MCA (Figure 3, Panel D).

The patient's neck wounds improved, and he was discharged from the neurosurgical ward to continue his treatment.

## Discussion

In this case report, CTA examination showed characteristic findings of a twig-like MCA anomaly. Diagnosis of this entity is a challenge due to the differential diagnosis with moyamoya angiopathy (MMA). The gold standard for diagnosis is digital subtraction angiography (DSA), which can demonstrate the classical plexiform network image that replaces the normal MCA trunk. Despite the major improvements in CT and magnetic resonance imaging (MRI) equipment, these vascular anomalies can easily mimic arterial occlusions. Treatment for tMCA can be either conservative or surgical, depending on the presence of hemorrhage, aneurysms, or massive hematomas [8,9].

It is very important to differentiate between tMCA and unilateral MMA, as the management strategies for these conditions differ significantly. A comparative study highlighted that tMCA cases exhibited no involvement of the internal carotid artery terminus or posterior cerebral artery and lacked transdural anastomoses, features commonly seen in MMA [10]. These distinctions are vital for accurate diagnosis and appropriate treatment planning. Thanks to advances in imaging, as in the case mentioned, the use of DSA remains the gold standard for diagnosing tMCA, providing detailed visualization of the vascular network. High-resolution MRI and magnetic resonance angiography (MRA) can also aid in diagnosis, particularly in distinguishing tMCA from other vascular anomalies. In cases where noninvasive imaging is inconclusive, DSA is essential for definitive diagnosis.

Recognizing tMCA is essential to avoid misdiagnosis and inappropriate interventions. Misinterpreting tMCA as an acute M1 occlusion could lead to unnecessary thrombolytic therapy or mechanical thrombectomy. Accurate diagnosis ensures that patients receive appropriate management and are spared from potential iatrogenic harm. In this case report, CTA examination showed characteristic findings of a twig-like MCA anomaly.

## Conclusions

This case report highlights a rare vascular anomaly that requires careful identification through radiological evaluation. With advancements in imaging technology, vascular abnormalities are frequently detected in asymptomatic patients, allowing prompt treatment. Understanding its pathophysiology and imaging features is fundamental for guiding safe and effective therapeutic decisions, particularly in patients requiring surgical intervention.
